# Identifying Group A Streptococcal Pharyngitis in Children Through Clinical Variables Using Machine Learning

**DOI:** 10.7759/cureus.37141

**Published:** 2023-04-04

**Authors:** Yoshifumi Miyagi

**Affiliations:** 1 Department of Pediatrics, Haibara General Hospital, Shizuoka, JPN

**Keywords:** chatgpt, auc, clinical symptoms, xgboost, machine learning (ml), group a streptococcal pharyngitis

## Abstract

Background

Group A Streptococcus (GAS) is the most common bacterial cause of pharyngitis in children. GAS pharyngitis requires antimicrobial agents, and rapid antigen detection tests (RADTs) are currently considered useful for diagnosis. However, the decision to perform the test is based on the pediatrician's examination findings, but the indicators are not clear. Therefore, we used machine learning (ML) to create a model to identify GAS pharyngitis from clinical findings and to explore important features.

Methods

ML with Python programming language was used for this study. Data from the included study involved 676 children aged 3 to 15 years diagnosed with pharyngitis, with positive results on the RADT serving as exposures, and negative results serving as controls. The ML performances served as the outcome. We utilized six types of ML classifiers, namely, logistic regression, support vector machine, k-nearest neighbor algorithm, random forest, an ensemble of them, Voting Classifier, and the eXtreme Gradient Boosting (XGBoost) algorithm. Additionally, we used SHapley Additive exPlanations (SHAP) values to identify important features.

Results

Moderately performing models were generated for all six ML classifiers. XGBoost produced the best model, with an area under the receiver operating characteristics curve of 0.75 ± 0.01. The order of important features in the model was palatal petechiae, followed by scarlatiniform rash, tender cervical lymph nodes, and age.

Conclusion

Through this study, we have demonstrated that ML models can predict childhood GAS pharyngitis with moderate accuracy using only commonly recorded clinical variables in children diagnosed with pharyngitis. We have also identified four important clinical variables. These findings may serve as a reference for considering indicators under the current guidelines recommended for selective RADTs.

## Introduction

Group A Streptococcus (GAS) is the most common cause of bacterial pharyngitis [1]. It is important to accurately diagnose bacterial pharyngitis since untreated group A streptococcal pharyngitis can lead to complications [2,3]. While the McIsaac score can be useful in differentiating GAS pharyngitis from clinical findings in children, the gold standard for diagnosing GAS pharyngitis is bacterial culture [4,5]. However, pharyngeal cultures take 24-48 hours and require culture equipment. To address these limitations, a rapid antigen detection test (RADT) has been developed that can detect group A Streptococcus in just a few minutes. Nevertheless, the decision to perform the test is currently based on the pediatrician's examination findings, but the indicators are not clear.

If all patients diagnosed with pharyngitis receive antimicrobials, the unnecessary administration of antimicrobials becomes problematic. On the other hand, testing all patients also raises the issue of cost-effectiveness. Guidelines from the American Heart Association, the Infectious Diseases Society of America, and the American Academy of Pediatrics recommend the use of RADTs selectively [6-8]. We believe that there is a need for an indicator to suggest the criteria for the indication for testing and antimicrobial therapy among patients diagnosed with pharyngitis. Thus, our objective was to develop a model to predict GAS pharyngitis and to identify important features in patients diagnosed with pharyngitis.

## Materials and methods

Data collection

As this study used publicly available supplemental data from previously published articles, Ethics Committee approval was not required. We utilized data from a French prospective multicenter cross-sectional study [9]. To our knowledge, this was the only published dataset available on GAS infections in children, and therefore, we used it for this study. The study included 676 children aged 3 to 15 years who were diagnosed with pharyngitis between October 1, 2010, and May 31, 2011. The exposure group consisted of 305 cases with a positive RADT (rapid immunochromatographic strip assay, Streptatest; Dectra Pharm, Eckbolsheim, France), while the control group included 371 cases with a negative RADT. The outcome of the study was to evaluate the performance of the machine learning (ML) classifiers.

Data preprocessing

The obtained dataset contained 17 characteristics, including age, sudden onset of sore throat, maximum body temperature (as reported by the accompanying parent), throat pain, cough, rhinorrhea, conjunctivitis, headache, pharyngeal erythema, tonsillar swelling, tonsillar exudate, palatal petechiae, nausea and/or vomiting, abdominal pain, diarrhea, tender cervical lymph nodes, and presence of a scarlatiniform rash. We also extracted one outcome variable, which was the result of RADT. For modeling purposes, attributes with missing values in the dataset were imputed using IterativeImputer [10]. The data was then standardized using StandardScaler [11]. Raw data was used for analysis with the eXtreme Gradient Boosting (XGBoost) algorithm, which does not require completion and standardization.

Data analysis

ML models were used to predict a positive or negative rapid test from the features in the data. Initially, logistic regression (LR), support vector machine (SVM), k-nearest neighbors (KNN) algorithm, and random forest (RF) were implemented. These hyperparameters were optimized using either GridSearchCV or Optuna [12,13]. Then, the four classifiers were combined using the Voting Classifier (VC) to ensemble them. Finally, the analysis was performed using XGBoost adjusted with Optuna.

Model development

Based on the hyperparameter results, LR was set to use C=0.1 and L2 penalty. For SVM, the hyperparameters were set to C=74.13 and linear kernel. For KNN, the number of clusters was set to 29 based on the best area under the receiver operating characteristics (ROC) curve (AUC). For RF, max_depth was set to 7, n_estimators to 80, and max_features to log2. In VC, the four classifiers were combined with soft voting and weights of (1, 1, 1, 1). For XGBoost, the following hyperparameters were selected: learning_rate = 0.1391, max_depth = 5, n_estimators = 39, min_child_weight = 9, gamma = 4.0015, subsample = 0.9325, and eval_metric = auc.

For LR, the model with L2 penalty is also called ridge regression, where L2 can reduce model complexity and prevent over-fitting, and C means inverse of regularization strength [14]. The kernel of SVM is the function used to determine the boundary surface, and in this study "linear" was chosen, where C stands for the regularization parameter [15]. For KNN, the value of k, meaning the number of clusters, was calculated from 0 to 100, and the number of clusters with the highest AUC was adopted. RF is a method classified as bagging in ensemble learning. max_depth, n_estimatars, and max_features are the depth of the tree, the number of trees, and the number of features to consider when looking for the best split, respectively [16]. As for XGBoost, learning_rate is a learning rate parameter to prevent overlearning. max_depth and n_estimators are the same as in RF. min_child_weight, gamma, and subsample are lower bounds on the weights of the leaves of the decision tree, the loss reduction due to adding leaves in the decision tree, and the fraction of random samples in each sample fraction randomly extracted in each decision tree, respectively [17]. SVM, RF, and XGBoost were tuned by Optuna. Optuna aims to efficiently search for parameters through Bayesian optimization, which means that it automatically searches for good parameters while keeping an eye on the search range [13].

Model evaluation and validation

Using a cross-validation approach with a 80/20 split for training and testing, the dataset was shuffled and a random seed of 42 was set. To evaluate the performance of the models, metrics such as accuracy, precision, recall, and F1-score were used. Additionally, ROC curves were plotted for each classifier, and the AUC was calculated to further assess the performance of the models.

Feature selection

Recursive feature elimination (RFE) was carried out for feature selection. Specifically, the boostRFE function of shap-hypetune was used [18].

Feature importance

Based on XGBoost tuned with Optuna, we confirmed the feature importance using SHapley Additive exPlanations (SHAP) values [19]. The SHAP value is a calculation of the impact of each characteristic variable on its predicted value. This allows us to visualize the impact of an increase or decrease in the value of a given characteristic variable.

Statistical analysis

The Python programming language, specifically version 3.7.12, was used for this study. A Mann-Whitney U-test was performed for all continuous variables because we found significant differences in the Shapiro-Wilk test, and a chi-square test was used for categorical variables.

## Results

Study participants

Of the 676 participants, 313 (46.3%) were girls and 363 (53.7%) were boys. All subjects had a median (interquartile range) age of 5.4 (4.2-7.2) years, and a median max temperature of 39.0 (38.2-39.4)°C. There was a significant difference in age between cases and controls (p < 0.05), but temperature was not significant (p = 0.12) (Table 1).

**Table 1:** Characteristics of this study All values are presented before imputation. Age and temperature are reported as median (interquartile range). Categorical variables are presented with the total number as the denominator and the number of findings as the numerator. Ratios are reported in parentheses as percentages.

	Total (N=676)	Case (N=305)	Control (N=371)	p
Continuous variables				
Age (years)	5.4 (4.2-7.2)	5.7 (4.5-7.3)	5.1 (4.0-7.0)	<0.05
Temperature	39.0 (38.2-39.4)	39.7 (38.0-39.1)	39.0 (38.3-39.5)	0.12
Categorical variables				
Petechiae	116/634 (18.3%)	86/283 (30.4%)	30/351 (8.5%)	<0.05
Scarlet	69/666 (10.4%)	59/302 (19.5%)	10/364 (2.7%)	<0.05
Tender	131/648 (20.2%)	80/292 (27.4%)	51/356 (14.3%)	<0.05
Abdominal pain	224/667 (33.6%)	97/300 (32.3%)	127/367 (34.6%)	0.59
Exudate	144/666 (21.6%)	55/299 (18.4%)	89/367 (24.3%)	0.08
Nausea/vomit	152/664 (22.9%)	75/298 (25.2%)	77/366 (21.0%)	0.24
Pain	542/644 (84.2%)	256/296 (86.5%)	286/348 (82.2%)	0.17
Cough	262/664 (39.5%)	94/297 (31.6%)	168/367 (45.8%)	<0.05
Tonsillar swelling	477/658 (72.5%)	224/298 (75.2%)	253/360 (70.3%)	0.19
Headache	186/662 (28.1%)	84/297 (28.3%)	102/365 (27.9%)	0.99
Erythema	599/672 (89.1%)	280/304 (92.1%)	319/368 (86.7%)	<0.05
Sudden	537/669 (80.3%)	249/302 (82.5%)	288/367 (78.5%)	0.23
Rhinorrhea	252/673 (37.4%)	101/304 (33.2%)	151/369 (40.9%)	<0.05
Diarrhea	33/663 (5.0%)	9/296 (3.0%)	24/367 (6.5%)	0.06
Conjunctivitis	19/671 (2.8%)	6/303 (2.0%)	13/368 (3.5%)	0.33

Model performance

**Figure 1:**
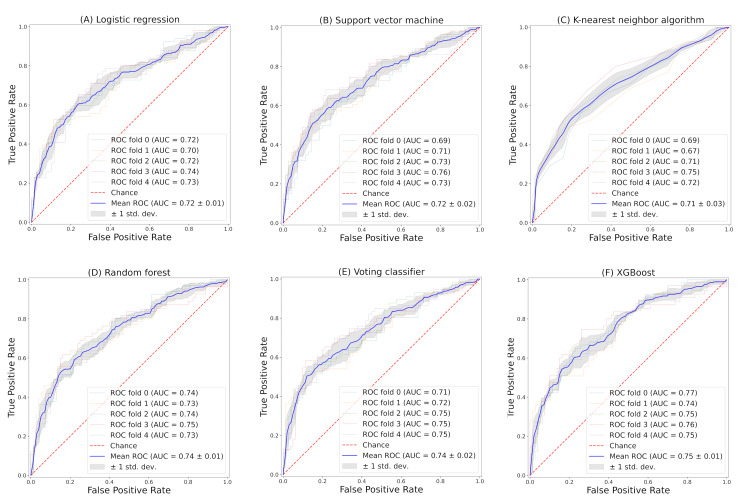
ROC curve for predicting GAS pharyngitis based on machine learning models XGBoost, eXtreme Gradient Boosting; ROC, receiver operating characteristics; AUC, area under the ROC curve; GAS, group A Streptococcus

Table 2 shows the accuracy, precision, recall, F-measure, and AUC for the six classifiers. For AUC, all classifiers showed moderate performance, ranging from 0.71 to 0.75 (Figures 1A-1F, Table 2), with XGBoost having the highest AUC of 0.75 ± 0.01 (Figure 1F).

**Table 2:** Performance evaluation of machine learning classifiers LR, logistic regression; SVM, support vector machine; KNN, k-nearest neighbors; RF, random forest; VC, Voting Classifier; XGBoost, eXtreme Gradient Boosting; AUC, area under the receiver operating characteristics curve; SD, standard deviation

	Accuracy	Precision	Recall	F-measure	AUC (mean ± SD)
LR	0.6849	0.7170	0.4984	0.5846	0.72 ± 0.01
SVM	0.6820	0.6673	0.5859	0.6235	0.72 ± 0.02
KNN	0.6554	0.6716	0.5016	0.5634	0.71 ± 0.03
RF	0.6864	0.7128	0.4689	0.6012	0.74 ± 0.01
VC	0.6908	0.7099	0.5475	0.6138	0.74 ± 0.02
XGBoost	0.6953	0.7043	0.5541	0.6166	0.75 ± 0.01

Feature selection

**Figure 2:**
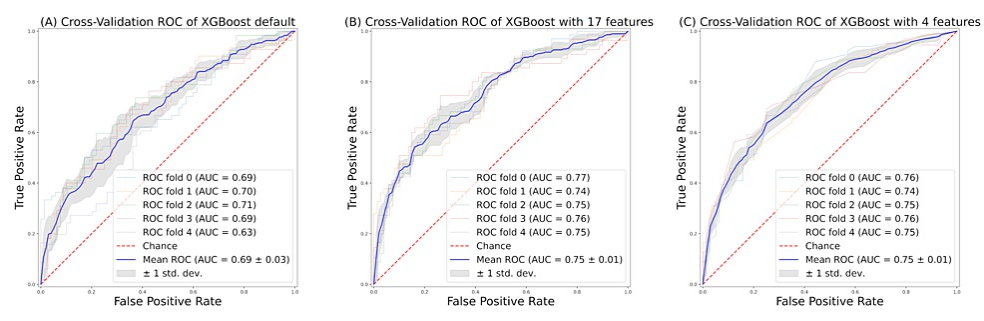
ROC curve for predicting GAS pharyngitis based on XGBoost: (A) default settings, (B) settings after parameter tuning with 17 features, and (C) settings after parameter tuning with 4 features reduced by RFE XGBoost, eXtreme Gradient Boosting; ROC, receiver operating characteristics; AUC, area under the ROC curve; GAS, group A Streptococcus; RFE, recursive feature elimination

We proceeded with the analysis based on the best-performing XGBoost, which had an AUC of 0.69 in its default configuration (Figure 2A). However, after tuning with Optuna, the AUC increased to 0.75 (Figure 2B). For feature selection, we used RFE to narrow down from 17 features to 4 features. The AUC remained at 0.75 in that model as well (Figure 2C).

Feature importance

**Figure 3:**
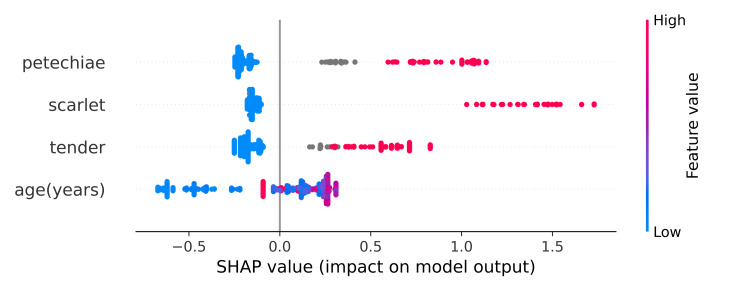
Feature importance using SHAP values Each point on the chart is one SHAP value for a prediction and feature. Red color means a higher value of a feature. Blue means a lower value of a feature. We can get the general sense of features’ directionality impact based on the distribution of the red and blue dots. SHAP, SHapley Additive exPlanations

The order of the most important features, as determined by SHAP values, was palatal petechiae, followed by scarlatiniform rash, tender cervical lymph nodes, and age, as shown in Figure 3.

## Discussion

Six ML classifiers were used to create models that could predict RADT positivity with moderate accuracy among patients diagnosed with pharyngitis. Among them, XGBoost retained the best performance. In addition, feature selection allowed the four most important factors in identifying GAS pharyngitis that were petechiae, followed by scarlatiniform rash, tender cervical lymph nodes, and age. The following discussion is based on the limited literature available.

The AUC is a commonly used metric for evaluating the accuracy of diagnostic tests and prediction models. Several studies have reported AUC values for the McIsaac scores in diagnosing GAS pharyngitis. McIsaac did not report an AUC for his original data, but a study using retail health data for patients aged three years and older reported an AUC of 0.71 [4,5,20]. A meta-analysis of eight studies using the McIsaac score found that the area under the summary receiver operating characteristic curve was 0.71 for McIsaac [21]. In this study, we used feature selection with RFE to reduce the 17 features to 4 while maintaining ML performance. The existing score has five features. In contrast, the best classifier in this study achieved an AUC of 0.75 with a reduction of one feature, suggesting that it may perform slightly better than conventional scoring systems. However, identifying new features that could further improve classifier performance remains a topic for future research.

XGBoost is an ensemble ML method known for its superior performance compared to other ML methods and was used to determine feature importance [22]. SHAP is a unified framework for interpreting predictions proposed in 2017 and is the only consistent and locally accurate feature attribution method based on expectations [23]. Feature importance represents how useful the input features are in predicting the target variable. Using the SHAP summary plot, we can observe two advantages: feature ranking and the effect of each feature. The most important finding in the present study was palatal petechiae, which has been reported in existing logistic regression evaluations with odds ratios of 3.18 to 9.32 for palatal petechiae in GAS pharyngitis [10,24]. Scarlatiniform rash, cervical lymph node tenderness, and age were also found to be important factors in identifying GAS pharyngitis in children diagnosed with pharyngitis. We suggest that GAS pharyngitis should be considered in patients with pharyngitis who have these findings, and that it may be indicated for antibiotics without testing.

The present study was conducted with subjects diagnosed with pharyngitis by a physician who were selected for the study. In order to prevent GAS sequelae, it is important to identify GAS pharyngitis without missing it. One option is to conduct a full case test or prescribe a full course of antibacterial drugs. However, if all patients were given antimicrobials, unnecessary use of antibiotics would occur. On the other hand, a report has recommended testing for all cases [10]. If all patients are tested, the question of cost-effectiveness arises. Therefore, an indicator is necessary to make an appropriate judgment based on reasonable indicators from among the clinical diagnoses. Guidelines also recommend the selective use of RADTs [6-8]. We also think similarly due to reasons such as time, cost, patient pain, unnecessary tests and antibiotics. At the very least, there is a high need for selective antibiotics now that they are recommended. Although it is important to include the cohort of patients who were not tested, we cannot mention that point. Nonetheless, we believe that this study, using a cohort of patients diagnosed with pharyngitis by a physician, is meaningful in order to create an indicator for appropriate antimicrobial administration or RADT. Based on these results, we propose an indicator to administer antibiotics without testing if pharyngitis is predicted as GAS pharyngitis in this ML model. However, further research is needed to confirm this.

The strength of this study is that it demonstrated the potential of utilizing ML algorithms to identify GAS pharyngitis from clinical findings. Additionally, this study was able to provide a non-invasive perspective by reusing previously published data. However, there are several limitations that need to be acknowledged. Firstly, a positive RADT result does not necessarily indicate pure GAS pharyngitis. Moreover, the present study included patients diagnosed with pharyngitis by a clinician. It may include a form of selection bias. If the goal is to diagnose GAS pharyngitis, it is important to include a cohort of patients who were not tested. The present study does not go that far. However, we believe that this study using a cohort of "patients diagnosed with pharyngitis by a physician" is also meaningful in creating an index for appropriate antimicrobial administration in an environment where a selective RADT is recommended.

## Conclusions

In conclusion, we have demonstrated that ML models can predict childhood GAS pharyngitis using only commonly recorded clinical variables in children diagnosed with pharyngitis. We have also identified four important clinical variables. These findings may serve as a reference for considering indicators under the current guidelines recommended for selective RADT. The challenge for the future is to search for new approaches to develop better-performing classifiers.
